# Histone deacetylase 2 is involved in DNA damage‐mediated cell death of human osteosarcoma cells through stimulation of the ATM/p53 pathway

**DOI:** 10.1002/2211-5463.12585

**Published:** 2019-01-30

**Authors:** Dan Sun, Meng Yu, Yuanyuan Li, Haotian Xing, Ying Gao, Zhihong Huang, Wenjun Hao, Kaining Lu, Chuize Kong, Osamu Shimozato, Toshinori Ozaki, Yuyan Zhu

**Affiliations:** ^1^ Department of Urology The First Hospital of China Medical University Shenyang China; ^2^ Department of Reproductive Biology and Transgenic Animal China Medical University Shenyang China; ^3^ Department of Molecular Medicine Life Science Institute Saga Medical Center KOSEIKAN Saga Japan; ^4^ Laboratory of DNA Damage Signaling Chiba Cancer Center Research Institute Chiba Japan

**Keywords:** adriamycin, ATM, cell death, DNA damage, HDAC2, p53

## Abstract

Tumor suppressor p53 is a short‐lived nuclear transcription factor, which becomes stabilized and activated in response to a wide variety of cellular stresses. Around 50% of human cancer tissues carry *p53* mutations, and certain *p53* mutations contribute to chemoresistance. In the present study, we found that histone deacetylase 2 (HDAC2) acts as a co‐activator of tumor suppressor p53 and participates in the early molecular events following DNA damage. Anti‐cancer drug adriamycin (ADR) treatment induced cell death in *p53*‐wild‐type human osteosarcoma U2OS cells, and this was accompanied by a remarkable accumulation of p53 and γH2AX. *HDAC2* gene silencing significantly decreased the sensitivity of U2OS cells to ADR and attenuated p53‐dependent DNA damage responses, such as ADR‐mediated phosphorylation of ataxia telangiectasia mutated (ATM) and p53, as well as accumulation of γH2AX and cleaved poly (ADP‐ribose) polymerase. However, *HDAC2* knockdown had a marginal effect on *p53*‐null human lung cancer H1299 cells following ADR exposure. In contrast, forced expression of HA‐HDAC2 promoted cell death and stimulated the transcriptional activity of p53. Moreover, p53 and HDAC2 were found to co‐precipitate with ATM. Together, our present results strongly suggest that the p53–HDAC2 axis plays a vital role in the regulation of the DNA damage response and also contributes to chemosensitivity of cancer cells.

**Abbreviations**
ADR: adriamycin
ATM: ataxia telangiectasia mutated
BAX: Bcl‐2‐associated X protein
DAPI: 4′,6‐diamidino‐2‐phenylindole
HDAC: histone deacetylase
PARP: poly (ADP‐ribose) polymerase
WST: water‐soluble tetrazolium salt

Tumor suppressor p53 is a short‐lived nuclear transcription factor, which becomes stabilized and activated in response to a wide variety of cellular stresses such as DNA damage, oxidative stress, oncogene activation, nutrient starvation and hypoxia 1, 2. Under normal conditions, p53 is kept at an extremely low level. Upon DNA insults, p53 is subjected to sequential post‐translational modifications including phosphorylation at Ser‐15 and acetylation at Lys‐373/382, which are catalyzed by ataxia telangiectasia mutated (ATM) and p300/CREB‐binding protein (CBP), respectively. These chemical modifications protect p53 from murine double minute 2 (MDM2)‐mediated ubiquitin–proteasome degradation. The activated form of p53 then triggers transient cell cycle arrest, cellular senescence and/or cell death dependent on the extent of DNA damage through the sequence‐specific transactivation of its downstream target genes such as *p21* ^*WAF1*^, *BAX*,* PUMA* and *NOXA* 1, 2. Extensive mutation searches demonstrated that around 50% of human cancer tissues carry *p53* mutations. Over 90% of *p53*'s mutations are detectable within its DNA‐binding domain 3. Thus, certain p53 mutants lack the sequence‐specific transactivation ability and contribute to the acquisition and/or maintenance of malignant phenotypes of cancer tissues such as serious chemoresistance, indicating that the sequence‐specific transcriptional activity of p53 is tightly linked to its tumor‐suppressive potential 4, 5. Moreover, mutant p53 exhibits a dominant‐negative behavior against wild‐type p53 as well as p53 family members such as TAp73 and TAp63. In addition to mutant p53, the transcriptional and pro‐apoptotic activity of wild‐type p53 is regulated by numerous cellular proteins including histone deacetylases (HDACs) 6.

Human HDACs have been divided into four groups (I, II, III and IV) based on their structure and subcellular localization 7. A growing body of evidence indicates that HDACs regulate a broad range of cellular processes through deacetylation of histones and non‐histone proteins 7, 8. Intriguingly, HDACs are dysregulated in a variety of human cancer tissues. For example, aberrant overexpression of class 1 HDACs (HDAC1, 2, 3 and 8) has been frequently detected in breast, prostate, colon, ovarian and pancreatic cancers 9. Indeed, it has been shown that HDAC2 overexpression correlates with a poor prognosis of breast cancer patients 10. Although it remains unclear how HDACs could contribute to carcinogenesis, certain HDAC inhibitors might be candidate anti‐cancer drugs. From a mechanistic point of view, Harms and Chen 11 demonstrated that *HDAC2* gene silencing inhibits cellular proliferation of breast cancer MCF‐7 cells bearing wild‐type *p53* through the activation of p53. Chen *et al*. 12 described that loss of HDAC1/2 causes the hyperacetylation of p53, its massive accumulation and enhanced cell death. Similarly, Brandl *et al*. 13 reported that HDAC2 binds to p53 and promotes its deacetylation at Lys‐320 in gastrointestinal cancer cells following DNA damage. These observations suggest that HDAC2 with a pro‐oncogenic potential prohibits p53 in certain cancer cells.

In the present study, we have focused on HDAC2 and sought to examine its possible impact on p53 in osteosarcoma U2OS cells following DNA damage. Unexpectedly, our present results strongly indicate that HDAC2 acts as a co‐activator of p53 through a collaboration with ATM.

## Materials and methods

### Cells and transfection

*p53*‐wild‐type human osteosarcoma U2OS and *p53*‐null human lung carcinoma H1299 cells were obtained from ATCC (Manassas, VA, USA), and maintained in Dulbecco's modified Eagle's medium supplemented with 10% heat‐inactivated FBS. For transfection, cells were transfected with the indicated siRNAs or with the expression plasmids using Lipofectamine 2000 reagent (Invitrogen, Thermo Fisher Scientific, Waltham, MA, USA) according to the manufacturer's instructions.

### RT‐PCR

Total RNA was isolated from cells using the RNeasy Mini Kit (Qiagen, Hilden, Germany) following the manufacturer's protocols. One microgram of total RNA was used to generate cDNA by the SuperScript VILO reverse transcription system (Invitrogen). Semi‐quantitative RT‐PCR was performed with GoTaq Green Master Mix (Promega, Madison, WI, USA) and gene‐specific primer sets. The housekeeping gene *GAPDH* was used as an internal control. Primer sequences and PCR conditions are available upon request.

### Immunoblotting

Cells were lysed in 1× SDS sample buffer supplemented with the protease inhibitor mixture (Sigma‐Aldrich, St Louis, MO, USA). Equal amounts of protein (30 μg) were separated on SDS/polyacrylamide gels and then transferred onto membrane filters (Merck Millipore, Amsterdam, the Netherlands). After blocking with 5% non‐fat dry milk, the membranes were probed with anti‐p53 (Santa Cruz Biotechnology, Dallas, TX, USA), anti‐phospho‐p53 at Ser‐15 (Cell Signaling Technology, Danvers, CA, USA), anti‐acetyl‐p53 at Lys‐373/382 (Upstate, Lake Placid, NY, USA), anti‐p21^WAF1^ (Santa Cruz Biotechnology), anti‐Bcl‐2‐associated X protein (BAX; Cell Signaling Technology), anti‐NOXA (Cell Signaling Technology), anti‐HDAC2 (Cell Signaling Technology), anti‐poly (ADP‐ribose) polymerase (PARP; Cell Signaling Technologies), anti‐γH2AX (BioLegend, San Diego, CA, USA), anti‐ATM (Santa Cruz Biotechnology), anti‐phospho‐ATM at Ser‐1981 (Merck Millipore) or with anti‐actin antibody (Santa Cruz Biotechnology) followed by an incubation with horseradish peroxidase‐conjugated secondary antibodies (Invitrogen). Immunodetection was performed with enhanced chemiluminescence (ECL; GE Healthcare Life Science, Piscataway, NJ, USA).

### Immunostaining

Cells were fixed in 3.7% formaldehyde for 30 min and permeabilized with 0.5% Triton X‐100 in PBS for 5 min at room temperature. After blocking with 3% BSA in PBS, cells were simultaneously incubated with anti‐HDAC2 and anti‐p53 antibodies for 1 h at room temperature. After washing in PBS, cells were incubated with fluorescent secondary antibodies (Invitrogen) for 1 h at room temperature. After washing in PBS, coverslips were mounted onto the slides using Vectashield (Vector Laboratories, Peterborough, UK). Cells were then examined under a confocal microscope (Leica, Milton Keynes, UK).

### Trypan blue assay

Twenty‐four hours after adriamycin (ADR) treatment, floating and adherent cells were collected and mixed with 0.4% trypan blue solution (Bio‐Rad Laboratories, Hercules, CA, USA) at room temperature for 2 min. Cells in the reaction mixtures were then counted with a TC‐20 automated cell counter (Bio‐Rad Laboratories). Trypan blue‐positive and ‐negative cells were considered to be dead and viable cells, respectively. All the experiments were performed in triplicate.

### FACS analysis

Twenty‐four hours after ADR exposure, floating and attached cells were harvested, washed in PBS and fixed in ice‐cold 70% ethanol. After fixation, cells were treated with 1 μg·mL^−1^ of propidium iodide and 1 μg·mL^−1^ of RNase A at 37 °C for 30 min in the dark. Cells were then analyzed by flow cytometry (FACSCalibur; BD Biosciences, San Jose, CA, USA).

### RNA interference

Negative control siRNA and siRNA against *HDAC2* (Santa Cruz Biotechnology) were introduced into U2OS cells at a final concentration of 10 nm. siRNA‐mediated knockdown of HDAC2 was verified by immunoblotting and RT‐PCR.

### Luciferase reporter assay

H1299 cells were transfected with the luciferase reporter construct carrying human *p21* ^*WAF1*^ or *NOXA* promoter, *Renilla* luciferase plasmid and a constant amount of p53 expression plasmid together with or without increasing amounts of the expression plasmid for HA‐HDAC2. Total amount of plasmid DNA per transfection was kept constant (510 ng) with pcDNA3. Forty‐eight hours after transfection, cell lysates were prepared and their luciferase activities were measured with a Dual‐Luciferase reporter assay system according to the manufacturer's suggestions (Promega).

### WST assay

Cells were transferred into 96‐well plates at a density of 1 × 10^3^ per well and incubated overnight. After the incubation, cells were exposed to the indicated concentrations of ADR. Twenty‐four hours after treatment, the relative number of viable cells was assessed by using Cell Counting Kit‐8 reagent (Dojindo, Kumamoto, Japan) according to the manufacturer's instructions. Cell Counting Kit‐8 (CCK‐8) contains water‐soluble tetrazolium salt (WST) and allows sensitive colorimetric assays for the determination of cell viability in cell proliferation and cytotoxicity assays. Experiments were performed in triplicate.

### Statistical analysis

Results were presented as mean ± SD of three independent experiments. Data were compared using one‐way ANOVA (ekuseru‐toukei 2010 software, Social Survey Research Information Co., Ltd, Tokyo, Japan), and a *P*‐value < 0.05 was considered significant.

## Results

### Expression level of HDAC2 remains largely unchanged during ADR‐mediated cell death of human osteosarcoma U2OS cells

**Figure 1:**
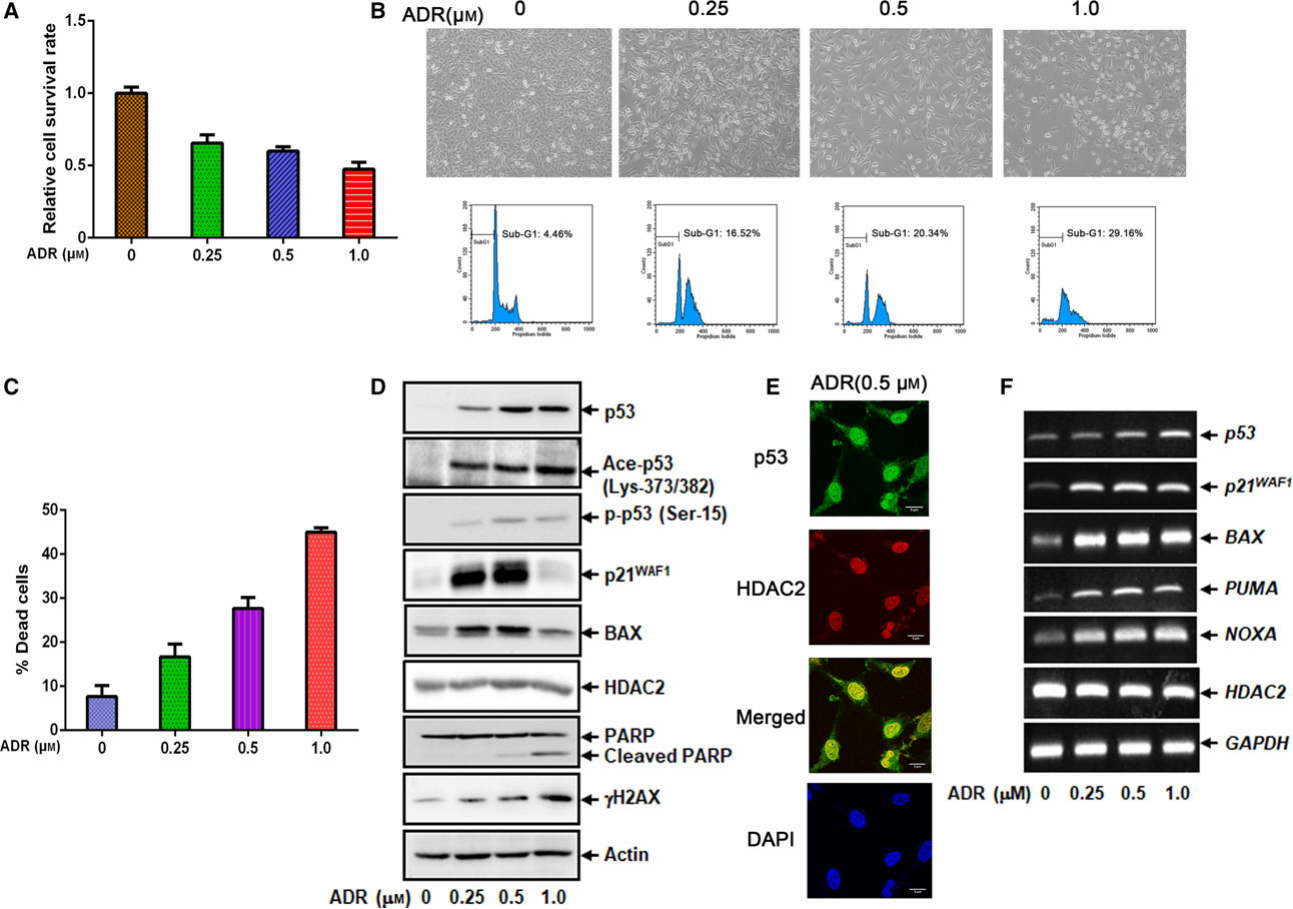
U2OS cells undergo cell death in response to ADR. (A) WST assay. U2OS cells were treated with the indicated concentrations of ADR. Twenty‐four hours after treatment, cell viability was assessed by WST assay. (B) FACS analysis. U2OS cells were exposed to the increasing concentrations of ADR. Twenty‐four hours after treatment, floating and attached cells were collected and analyzed by FACS. Representative microscopic images of these cells are indicated. (C) Trypan blue assay. U2OS cells were treated as in (A). Twenty‐four hours after treatment, floating and attached cells were harvested, mixed with 0.4% trypan blue solution and number of trypan blue‐positive cells (dead cells) was measured. (D) Immunoblotting. U2OS cells were treated as in (A). Twenty‐four hours after treatment, cell lysates were extracted and analyzed by immunoblotting. Actin was used as a loading control. (E) Indirect immunostaining. U2OS cells were exposed to ADR (0.5 μm). Twenty‐four hours after treatment, cells were fixed and simultaneously stained with anti‐p53 and anti‐HDAC2 antibodies. Cell nuclei were stained with 4′,6‐diamidino‐2‐phenylindole (DAPI). (F) RT‐PCR. U2OS cells were treated as in (A). Twenty‐four hours after treatment, total RNA was prepared and subjected to RT‐PCR. *GAPDH* was used as an internal control. All results shown are representative of at least three independent experiments. The error bars represent SD.

To determine whether there is a functional interaction between p53 and HDAC2 during DNA damage‐induced osteosarcoma cell death, we sought to examine the expression levels of p53, p53‐target gene products and HDAC2 in *p53*‐wild‐type human osteosarcoma U2OS cells exposed to increasing concentrations of ADR. As a result, U2OS cells underwent growth arrest and cell death following ADR treatment in a dose‐dependent manner (Fig. 1A–C). Under these experimental conditions, cell lysates were prepared and analyzed by immunoblotting. As seen in Fig. 1D, ADR‐mediated accumulation of γH2AX and cleaved PARP was detectable, indicating that U2OS cells receive DNA damage and then undergo cell death.

**Figure 2:**
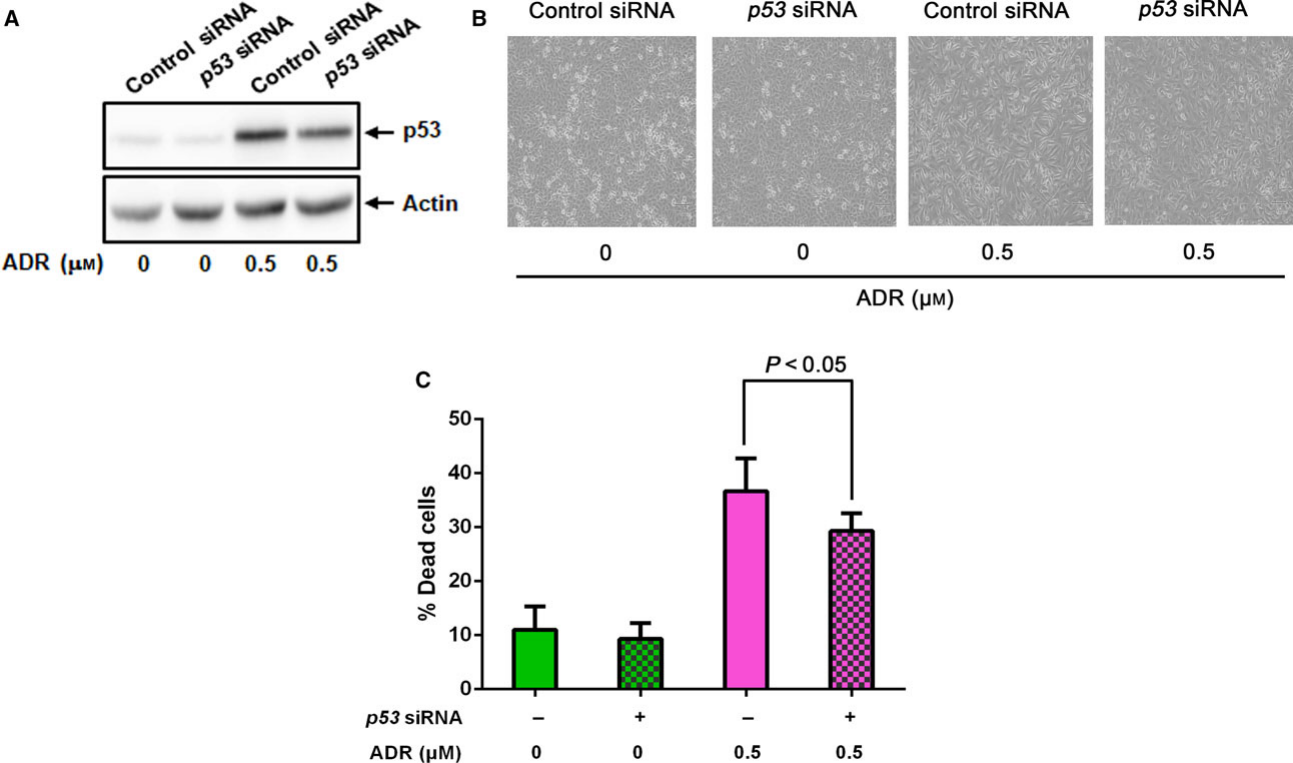
siRNA‐mediated *p53* knockdown attenuates ADR‐induced cell death in U2OS cells. (A) Western blot analysis. U2OS cells were transfected with control siRNA or with siRNA against *p53*. Twenty‐four hours after transfection, cells were treated with ADR (0.5 μm) or left untreated. Expression of p53 was detected by the indicated antibody. (B) Representative microscopic images of U2OS cells treated as in (A). (C) Trypan blue assay. U2OS cells were transfected and treated as in (A). Twenty‐four hours after treatment, floating and attached cells were harvested and subjected to trypan blue assay. All results represent at least three independent experiments. Data show mean ± SD (*n *=* *3, *P *<* *0.05). Data were compared using one‐way ANOVA.

**Figure 3:**
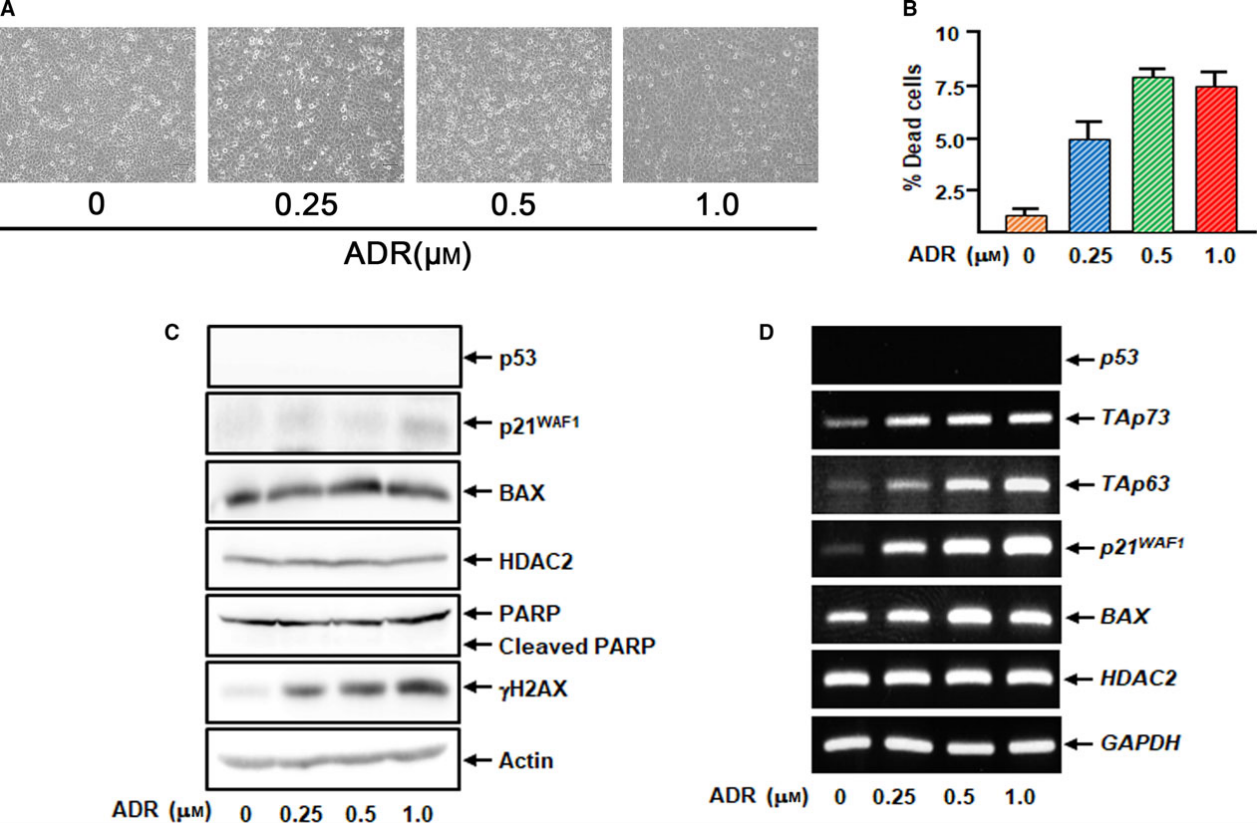
H1299 cells poorly respond to ADR. (A,B) H1299 cells were exposed to increasing concentrations of ADR. Twenty‐four hours after treatment, microscopic images of these cells were taken (A). Floating and attached cells were collected and analyzed by trypan blue assay (B). (C) Immunoblotting. H1299 cells were treated as in (A). Twenty‐four hours after treatment, cell lysates were extracted and analyzed by immunoblotting. Actin was used as a loading control. (D) RT‐PCR. H1299 cells were treated as in (A). Twenty‐four hours after treatment, total RNA was prepared and subjected to RT‐PCR. *GAPDH* was used as an internal control. All results represent at least three independent experiments. The error bars represent SD.

As described previously 14, ADR treatment resulted in a marked induction of p53 accompanied by its phosphorylation at Ser‐15 as well as acetylation at Lys‐373/382 (Fig. 1D). For p53‐target gene products, pro‐arrest p21^WAF1^ and pro‐apoptotic BAX were induced in response to ADR, while the amount of HDAC2 remained unchanged regardless of ADR exposure. During ADR‐mediated cell death, HDAC2 was co‐localized with p53 in the cell nucleus (Fig. 1E). RT‐PCR analysis demonstrated that p53‐target genes such as *p21* ^*WAF1*^, *BAX*,* PUMA* and *NOXA* are significantly up‐regulated following ADR exposure, whereas *HDAC2* transcription level is basically constant (Fig. 1F). Consistent with these observations, knockdown of *p53* in U2OS cells partially attenuated ADR‐mediated cell death (Fig. 2). In contrast, ADR treatment with the same increasing doses had a negligible impact on apoptotic cell death induction in *p53*‐null human lung carcinoma H1299 cells, although accumulation of γH2AX was detectable (Fig. 3A–C). p21^WAF1^ and BAX were mildly increased at the protein level in these cells, in spite of their marked induction at the mRNA level (Fig. 3C,D). Notably, the p53 family members p63 and p73 were also up‐regulated following ADR exposure, indicating that instead of p53, ADR‐induced p63 and p73 up‐regulated *p21* ^*WAF1*^ and *BAX* in *p53*‐null H1299 cells. Taken together, our results suggest that U2OS cells undergo cell death following ADR exposure at least in part in a p53‐dependent manner.

### HDAC2 gene silencing suppresses ADR‐mediated cell death of U2OS cells

**Figure 4:**
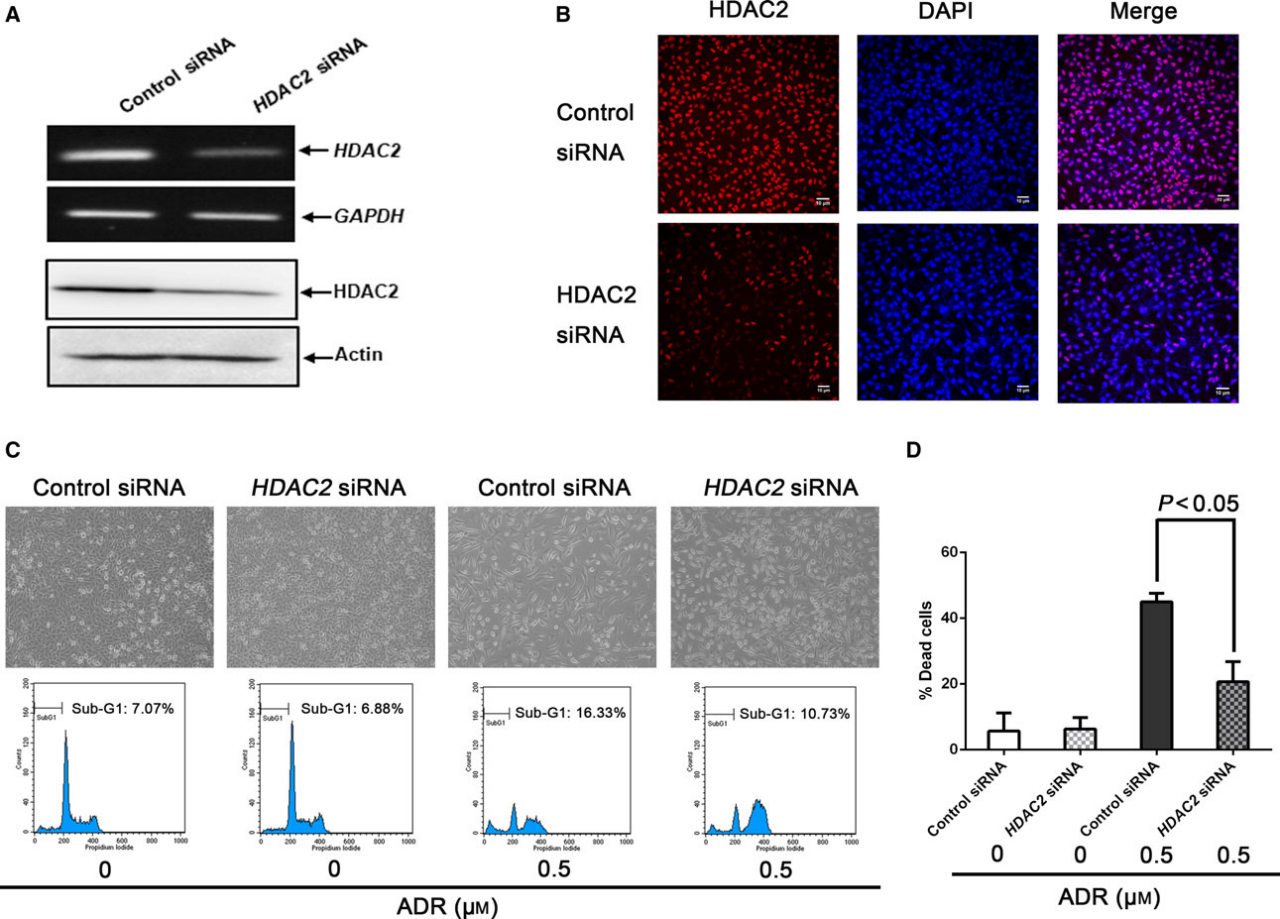
Knockdown of *HDAC2* attenuates ADR‐mediated cell death of U2OS cells. (A) The efficiency of siRNA‐mediated *HDAC2* knockdown. U2OS cells were transfected with control siRNA or with siRNA targeting *HDAC2*. Twenty‐four hours after transfection, total RNA and cell lysates were prepared and analyzed by RT‐PCR (upper) and immunoblotting (lower), respectively. (B) Indirect immunostaining. U2OS cells treated as in (A) were stained with anti‐HDAC2 antibody and DAPI. (C) FACS analysis. U2OS cells were transfected as in (A). Twenty‐four hours after transfection, cells were treated with ADR (0.5 μm) or left untreated. Twenty‐four hours after treatment, representative pictures were taken (upper). Floating and attached cells were harvested and analyzed by FACS (lower). (D) Trypan blue assay. U2OS cells were transfected and treated as in (C). Twenty‐four hours after treatment, floating and adherent cells were collected and subjected to trypan blue assay. All results shown are representative of at least three independent experiments. Data show mean ± SD (*n *=* *3, *P *<* *0.05). Data were compared using one‐way ANOVA.

To explore the role of HDAC2 during DNA damage‐induced cell death, U2OS cells were transfected with control siRNA or with siRNA targeting *HDAC2*. As shown in Fig. 4A,B, siRNA‐mediated knockdown of *HDAC2* significantly decreased HDAC2 at mRNA and protein levels. Under these experimental conditions, transfected cells were treated with ADR or left untreated. Twenty‐four hours after treatment, floating and attached cells were collected and subjected to FACS analysis and trypan blue assay. Close inspection of the upper panels of Fig. 4C revealed that ADR‐caused decrease in number of attached cells is partially restored by *HDAC2* silencing. In support of these observations, FACS analysis and trypan blue assay demonstrated that ADR‐mediated cell death is markedly attenuated in *HDAC2*‐depleted cells (Fig. 4C,D). Together, it is likely that HDAC2 plays a critical role in DNA damage‐mediated cell death.

### Depletion of HDAC2 impairs ADR‐stimulated induction and activation of p53

**Figure 5:**
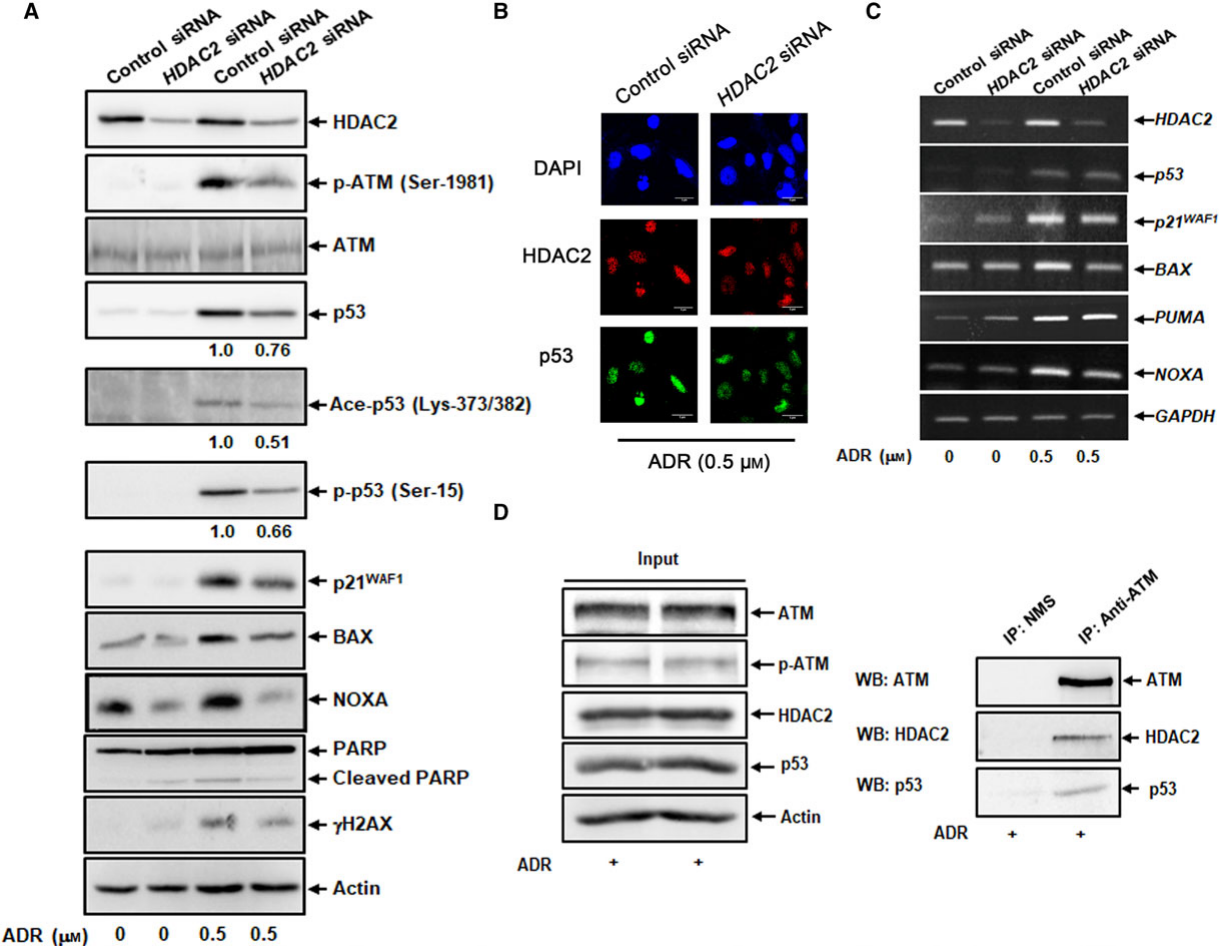
Depletion of *HDAC2* impairs ADR‐dependent activation of p53. (A) Immunoblotting. U2OS cells were transfected with control siRNA or with siRNA targeting HDAC2. Twenty‐four hours after transfection, cells were treated with ADR (0.5 μm) or left untreated. Twenty‐four hours after treatment, cell lysates were prepared and analyzed by immunoblotting. The ratios of p53, p‐p53 at Ser‐15 and Ace‐p53 at Lys‐373/382 in knocked down cells to total p53 in control cells as examined by densitometric analysis are also shown. (B) Indirect immunostaining. *HDAC2*‐knocked down U2OS cells were exposed to ADR (0.5 μm). Twenty‐four hours after treatment, cells were simultaneously stained with anti‐HDAC2 and anti‐p53 antibodies. Cell nuclei were stained with DAPI. (C) RT‐PCR. U2OS cells were transfected and treated as in (A). Twenty‐four hours after treatment, total RNA was extracted and analyzed by RT‐PCR. (D) Immunoprecipitation assay. U2OS cells were exposed to ADR (0.5 μm). Twenty‐four hours after treatment, cell lysates were immunoprecipitated with normal mouse serum or with monoclonal anti‐ATM antibody. The resultant immunoprecipitates were analyzed by immunoblotting with the indicated antibodies (lower); 1/20 of inputs are also shown (upper). All results shown are representative of at least three independent experiments.

**Figure 6:**
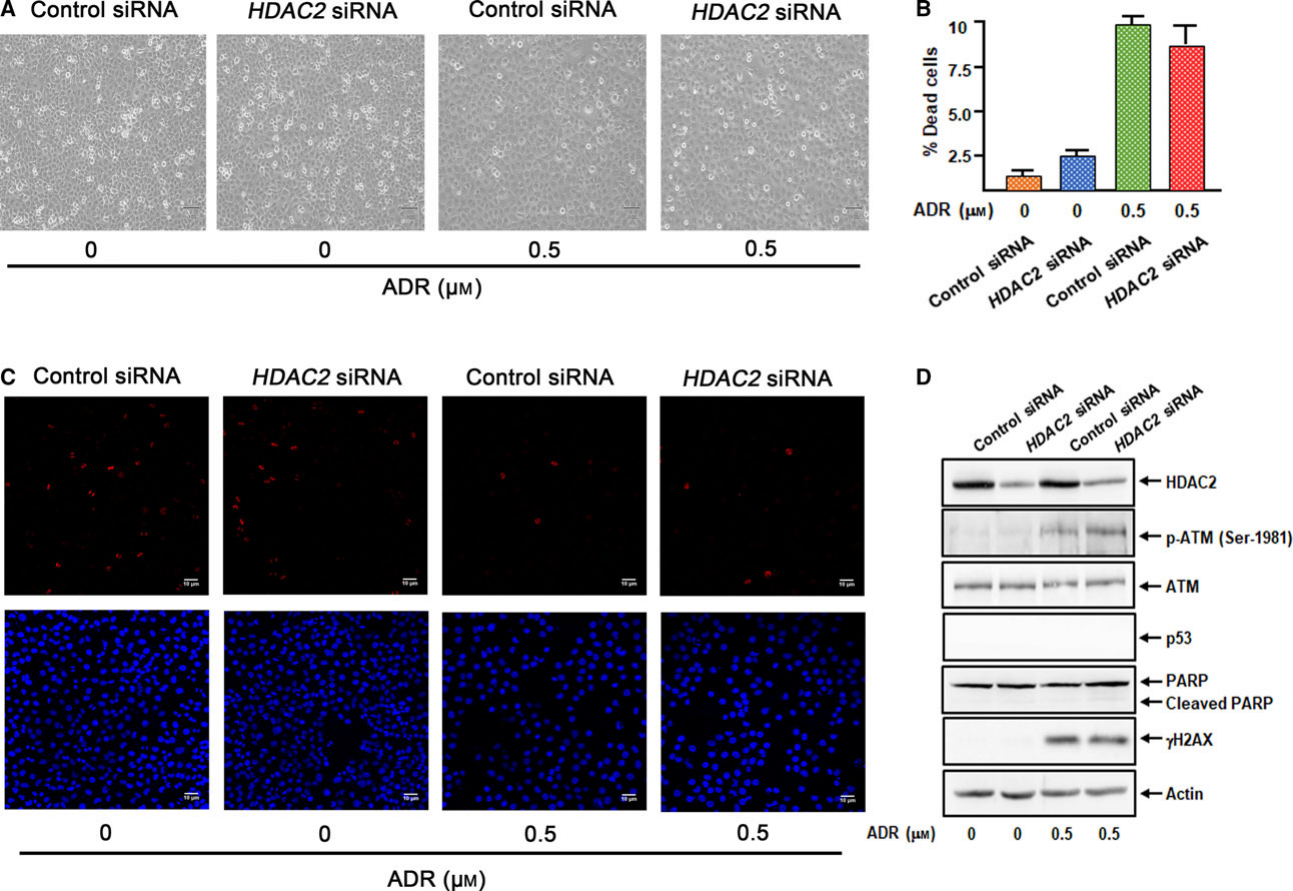
Depletion of *HDAC2* has an undetectable effect on ADR‐mediated growth retardation and cell death of H1299 cells. (A–C) H1299 cells were transfected with control siRNA or with siRNA against *HDAC2*. Twenty‐four hours after treatment, representative pictures were taken (A). Floating and adherent cells were collected and analyzed by trypan blue assay (B). Under the same experimental conditions, cells were stained with anti‐Ki67 antibody and DAPI (C). (D) Expression analysis. H1299 cells were transfected and treated as in (A). Twenty‐four hours after treatment, cell lysates were prepared and analyzed by immunoblotting with the indicated antibodies. All results shown are representative of at least three independent experiments. The error bars represent SD.

Considering that HDAC2 is co‐localized with p53 in the cell nucleus following ADR exposure, it is possible that there could exist a functional interaction between HDAC2 and p53 in response to ADR. To address this issue, we checked the expression levels of p53 and p53‐target gene products in *HDAC2*‐depleted U2OS cells exposed to ADR. Notably, *HDAC2* gene silencing resulted in a marked suppression of ADR‐induced accumulation, phosphorylation (Ser‐15) and acetylation (Lys‐373/382) of p53 (Fig. 5A). Down‐regulation of p53 in *HDAC2*‐knocked down cells was also confirmed by indirect immunostaining experiments (Fig. 5B). Moreover, ADR‐dependent phosphorylation of ATM, accumulation of γH2AX and cleavage of PARP was obviously prohibited in *HDAC2*‐knocked down U2OS cells (Fig. 5A). Consistent with these results, ADR‐mediated up‐regulation of p53‐target gene products such as p21^WAF1^, BAX and NOXA was markedly attenuated at both mRNA and protein levels in *HDAC2*‐depleted U2OS cells (Fig. 5A,C). ADR‐caused increase in the amount of *PUMA* was unaffected by *HDAC2* depletion. On the other hand, endogenous HDAC2 forms a complex with p53 and ATM in ADR‐treated U2OS cells (Fig. 5D). By contrast, *HDAC2* gene silencing in H1299 cells had no significant effect on ADR‐mediated cell death and growth retardation, as indicated by the results of a trypan blue assay and Ki‐67 immunostaining, respectively (Fig. 6A–C), and had a marginal effect on ADR‐dependent phosphorylation of ATM as well as accumulation of γH2AX (Fig. 6D).

**Figure 7:**
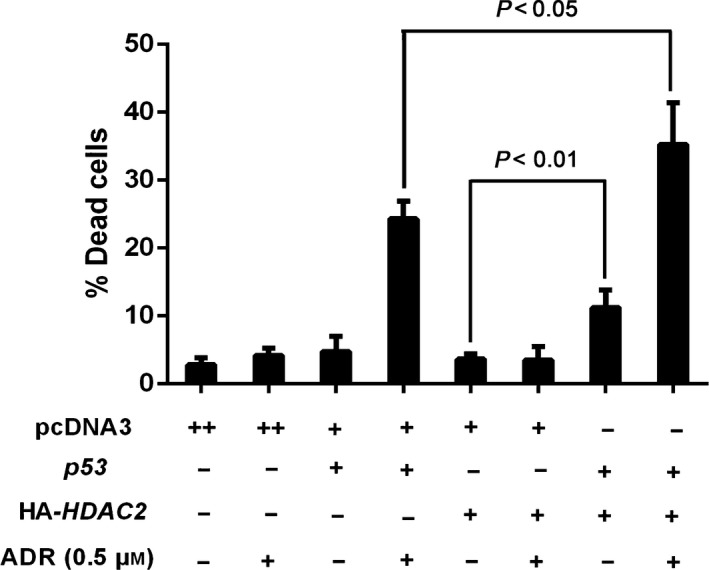
Cell death‐potentiating effect of HDAC2 depends on p53. H1299 cells were transfected with the indicated combinations of the expression plasmids. Twenty‐four hours after transfection, cells were treated with or without 0.5 μm of ADR. Twenty‐four hours after treatment, floating and attached cells were harvested and analyzed by trypan blue assay. All results shown are representative of at least three independent experiments. Data show mean ± SD (*n *=* *3, *P *<* *0.05 or *P *<* *0.01). Data were compared using one‐way ANOVA.

These observations suggest that the ADR‐induced cell death‐potentiating effect of HDAC2 might be dependent on p53. To check this possibility, H1299 cells were transfected with the indicated combinations of the expression plasmids and then treated with or without ADR. As clearly seen in Fig. 7, HDAC2 alone had an undetectable effect on H1299 cells in the presence or absence of ADR, whereas HDAC2 had the ability to markedly stimulate cell death in H1299 cells expressing the exogenous p53. Together, these results strongly indicate that HDAC2 potentiates p53‐dependent cell death pathway in response to DNA damage, and its effect depends on p53.

### Forced expression of HDAC2 increases ADR‐induced cell death and enhances transcriptional activity of p53

**Figure 8:**
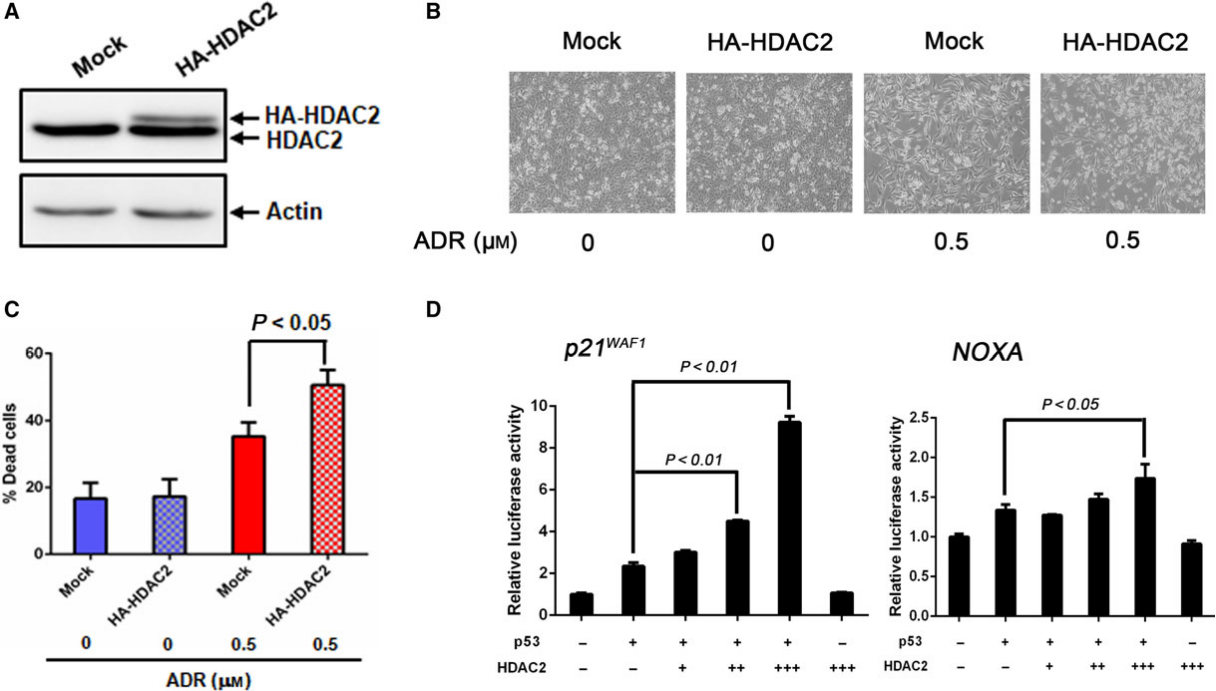
HDAC2 increases ADR‐induced cell death and enhances the transcriptional activity of p53. (A) Forced expression of HA‐HDAC2. U2OS cells were transfected with the empty plasmid or with the expression plasmid for HA‐HDAC2. Forty‐eight hours after transfection, cell lysates were analyzed by immunoblotting with anti‐HDAC2 (upper) or with anti‐actin (lower) antibody. (B) Representative microscopic images of HDAC2‐overexpressed U2OS cells in response to ADR. U2OS cells were transfected with the empty plasmid or with the expression plasmid for HA‐HDAC2. Twenty‐four hours after transfection, cells were exposed to ADR (0.5 μm). Twenty‐four hours after treatment, photos were taken. (C) Trypan blue assay. Floating and attached cells in (B) were harvested and mixed with 0.4% trypan blue solution and the number of trypan blue‐positive cells (dead cells) was measured. All results shown are representative of at least three independent experiments. Data show mean ± SD (*n *=* *3, *P *<* *0.05). (D) Luciferase reporter assay. H1299 cells were transfected with the luciferase construct bearing *p21* ^*WAF*^ ^*1*^ or *NOXA* promoter, *Renilla* luciferase plasmid and a constant amount of p53 expression plasmid plus increasing amounts of HA‐HDAC2 expression plasmid. Forty‐eight hours after transfection, cell lysates were prepared and their luciferase activities driven by *p21* ^*WAF*^ ^*1*^ (*n *=* *3, *P *<* *0.01) or *NOXA* (*n *=* *3, *P *<* *0.05) promoter were measured. All results shown are representative of at least three independent experiments. Data were compared using one‐way ANOVA.

To further confirm the above‐mentioned hypothesis, we sought to examine whether ectopic expression of HDAC2 could promote ADR‐induced cell death. To this end, U2OS cells were transfected with the HA‐HDAC2 expression vector and then treated with ADR or left untreated. Twenty‐four hours after treatment, cell death was evaluated by trypan blue exclusion assay. The expression efficiency of HA‐HDAC2 was verified by immunoblotting (Fig. 8A). As expected, ectopic expression of HDAC2 increased ADR‐induced cell death in U2OS cells (Fig. 8B,C). Next, we investigated the effect of HDAC2 on transcriptional activity of p53. For this purpose, *p53*‐null H1299 cells were transfected with a constant amount of p53 expression plasmid together with or without increasing amounts of the expression plasmid for HA‐HDAC2, along with luciferase reporter vector driven by *p21* ^*WAF1*^ or *NOXA* promoter. Forty‐eight hours after transfection, cell lysates were prepared and their luciferase activities were measured. Under our experimental conditions, luciferase reporter bearing *p21* ^*WAF1*^ or *NOXA* promoter responded to the exogenously expressed p53, and ectopic expression of HA‐HDAC2 remarkably enhanced p53‐mediated increase in luciferase activity driven by *p21* ^*WAF1*^ or *NOXA* promoter, whereas HA‐HDAC2 expression alone had no effect on their activities (Fig. 8D). Collectively, it is suggestive that HDAC2 has an ability to stimulate transcriptional activity of p53.

## Discussion

In the present study, we have found that *HDAC2* gene silencing attenuates the ATM/p53‐mediated cell death pathway of osteosarcoma U2OS cells in response to ADR, indicating that HDAC2 participates in the early molecular events of DNA damage response, and also acts as a co‐activator of p53.

Based on our current results, depletion of *HDAC2* decreased ADR sensitivity of U2OS cells bearing wild‐type *p53*. In contrast, *p53*‐null H1299 cells poorly responded to ADR, and knockdown of *HDAC2* had a negligible effect on their response rate to ADR. Under our experimental conditions, it is likely that HDAC2 might function in a p53‐dependent manner. Notably, knockdown of *HDAC2* in U2OS cells significantly inhibited ADR‐mediated accumulation of p53, its phosphorylation at Ser‐15 and its acetylation at Lys‐373/382. Since these modifications stimulate the transcriptional as well as pro‐apoptotic activity of p53 1, 2, ADR‐induced p53‐target gene expression such as *p21* ^*WAF1*^, *BAX* and *NOXA*, and cleavage of PARP was markedly prohibited in *HDAC2*‐knocked down U2OS cells. Consistent with these results, forced expression of HA‐HDAC2 promoted ADR‐induced cell death and augmented p53‐dependent transactivation of *p21* ^*WAF1*^ and *NOXA* promoters as examined by luciferase reporter assays. Thus, our present observations suggest that HDAC2 is required at least in part for p53‐dependent cell death in response to DNA damage. In support of this notion, Thurn *et al*. 15 described that HDAC inhibitor suberoylanilide hydroxamic acid, which shows limited clinical activity to solid tumors, reduces p53 activity in response to DNA damage. Additionally, Lebrun‐Julien and Suter found that the simultaneous ablation of *HDAC1/2* protects neuronal cell death *in vivo* through the impairment of p53‐dependent transactivation of *p21* ^*WAF1*^ as well as *PUMA* 16.

In contrast to our present results, mounting evidence indicates that HDAC2 is aberrantly overexpressed in a variety of cancer tissues and implicated in the promotion of malignant phenotypes of certain cancer cells such as anti‐cancer drug resistance. For example, Wagner *et al*. 17 described that higher expression level of HDAC2 is closely linked to cisplatin resistance of colon cancer cells. Fritsche *et al*. 18 revealed that HDAC2 contributes to the resistance to etoposide of pancreatic cancer cells. In addition, Jung *et al*. 19 reported that inactivation of HDAC2 stimulates p53‐dependent cell death of lung cancer cells. In accordance with these observations, Harms and Chen 11 found that knockdown of *HDAC2* in breast cancer cells enhances the transcriptional activity of p53. Meanwhile, Seo *et al*. 20 described that the elevated expression of HDAC2 results in better prognosis of estrogen receptor (ER)‐negative breast cancer patients. Recently, Chaiyawat *et al*. 21 demonstrated that a decrease in the expression level of HDAC2 is associated with poor prognosis of osteosarcoma patients. These findings indicate that HDAC2 might function in a cellular context‐dependent manner. Further studies are required to adequately address the functional as well as clinical significance of HDAC2.

Another finding of the present study was that *HDAC2* gene silencing reduces ADR‐mediated phosphorylation of ATM, phosphorylation of p53 at Ser‐15 and accumulation of γH2AX in U2OS cells. Since ATM‐mediated phosphorylation of histone H2AX is one of the early molecular events during the DNA damage response and p53 is the substrate of ATM for the propagation of DNA damage signaling, it is likely that HDAC2 participates in the activation of ATM in U2OS cells. Under our experimental conditions, the anti‐ATM immunoprecipitates contained p53 and HDAC2, indicating that ATM forms a complex with p53 and HDAC2. Previously, Khanna *et al*. 22 described that ATM directly binds to p53 and mediates its phosphorylation at Ser‐15. While *HDAC2* depletion had a negligible effect on ADR‐mediated phosphorylation of ATM in H1299 cells, it is likely that the effect of HDAC2 on ATM depends on p53. In support of our results, Thurn *et al*. 15 found that knockdown of *HDAC1/2* abrogates DNA damage‐mediated activation of ATM and reduces the stimulation of certain p53‐target gene expression in *p53*‐wild‐type breast cancer cells. Intriguingly, Kobayashi *et al*. 23 demonstrated that γH2AX directly interacts with ATM and enhances its kinase activity to amplify DNA damage. Additionally, Tanaka *et al*. 24 described that p53 plays a vital role in facilitating phosphorylation of H2AX. At present, we do not know the precise molecular mechanisms of how HDAC2 could regulate the DNA damage‐induced ATM/p53 cell death pathway. Since HDAC2 has an ability to deacetylate its substrate proteins, it is important to identify key substrate(s) of HDAC2 during the DNA damage response.

Together, our current results strongly suggest that HDAC2 acts as a co‐activator of p53 in response to DNA damage, and might provide a clue to develop a novel strategy to improve chemosensitivity.

## Author contributions

YZ and TO conceived and designed the project; DS, MY and TO obtained the data; DS, TO, YL, HX, YG, ZH, WH, KL, CK and OS analyzed and interpreted the data; and TO, YL and YZ wrote the paper.

## Conflict of interest

The authors declare no conflict of interest.
